# Supplementary material for: Exploring the Mental, Social, and Lifestyle Effects of a Positive COVID-19 Infection on Syrian Refugees in Jordan: A Qualitative Study
Source: Int J Environ Res Public Health. 2022 Oct 2;19(19):12588. doi: 10.3390/ijerph191912588 (PMC9566814; doi:10.3390/ijerph191912588)
Supplement: Supplementary file 1 [file ijerph-19-12588-s001.zip › ijerph-1707271-supplementary.pdf]

Supplementary Table S1. Interview Guide.

Participant	Age (years)	Gender	Education	Occupation	Marital status	Place of residence	Co-habitants	Arrival date to Jordan	Financial source
Refugee01F	56	Female	School	Housewife	Married	Irbid-outside camp	Daughters and husband	2012, 3 years later left the camp to Irbid rented house	A 50 Jordanian Dinars (JDs) monthly food coupon from UNHCR, approximate salary by her husband around 200 JDS
Refugee 01M	31	Male	High school	Ex-mechanic	Single	Irbid-outside camp	Five single brothers	2012 inside camp, then moved to Irbid rented house	My five brothers working to cove life needs approximate income between 500-700 JDS
Refugee 02F	51	Female	University /bachelor degree in law	Housewife	Married	Irbid-outside camp	Husband, son and daughter	2015 came to Amman then moved to Irbid rented house	Husband working with good salary approximate salary around 200 JDS and 300 JDS
Refugee 02M	25	Male	Studying the English language at a private university	Waiter	Single	Irbid-outside camp	Parents, one sister and one brother	2012 stayed at camp for one year then moved to Irbid rented house	Father is working as a private English teacher, and mother sells homemade pastries<br>Approximate<br>Income around 250 JDS and 350 JDS
Refugee 03F	25	Female	University /bachelor degree in Finance	Secretory	Single	Irbid-outside camp	Live with parents and two brothers	2012, then moved from camp to rented house in Irbid at 2015	Father is the driver, and I am secretory<br>Approximate income around 250 and 350 JDS
Refugee 03M	45	Male	School	Casher	Married	Irbid-outside camp	Live with wife and three children	In 2013 direct to Irbid rented house	50 jds monthly food coupon from UNHCR<br>Approximate salary between 150 and 200 JDS
Refugee 04F	45	Female	High school	Housewife	Married	Irbid-outside camp	Live with husband and daughter	2012, then moved from camp to rented house in Irbid 6 months later	Husband is working as a porter and receiving a monthly 50jds food coupon from UNHCR<br>Approximate income 150 JDS
Refugee 04M	49	Male	High school	Painter	Married	Irbid-outside camp	Live with wife, son and two daughters	2013, then moved from camp to rented house in Irbid a few months later	Only work is the financial source<br>Approximate income 150 to 250 JDS
Refugee 05F	20	Female	Studying nutrition at university	Student	Single	Irbid-outside camp	With mother and brother	Came from Turkey in 2016 and bought a house in Irbid	Our financial status is perfect<br>Approximate income more than 500 JDS per month
Refugee 05M	39	Male	High school	Porter	Married	Irbid-outside camp	With wife and daughter	Lived in al Zaatari camp from 2012 to 2015 then moved to a rented house in Irbid	Approximate income between 150 to 200 JDS

**Refugee 06F**	38	Female	Bachelor degree in management	Receptionist	Married	Almafraq-inside camp	Husband 3 sons and three daughters	Almafraq-inside camp since 2012	I received a monthly 50 JDS food coupon and 20JD for each one of my family members by UNCHR in addition to my job<br>Approximate income 260 JDS
**Refugee 06M**	37	Male	Diploma of management	Security guard	Married	Almafraq-inside camp	With wife	Almafraq-inside camp since 2014	I am working, and we are receiving a monthly 50 JDS food coupon and 20JD for each one of family members by UNCHR<br>Approximate income 140 JDS
**Refugee 07F**	42	Female	High school	Housewife	Married	Almafraq-inside camp	With husband and three sons and two daughters	Almafraq-inside camp since 2013	Are receiving a monthly 50 JDS food coupon and 20JD for each one of a family member by UNCHR<br>Approximate income 140 JDS
**Refugee 07M**	43	Male	Diploma of nursing	Coordinator of the clinic inside the camp	Married	Almafraq-inside camp	With a wife and four daughters	Almafraq-inside camp since 2012	Receiving a monthly 50 JDS food coupon and 20JD for each one of a family member by UNCHR<br>Approximate income 220 JDS
**Refugee 08F**	29	Female	High school	Distribution of health brochures	Married	Almafraq-inside camp	With husband and one son and two daughters	Almafraq-inside camp since 2014	Are receiving a monthly 50 JDS food coupon and 20JD for each one of a family member by UNCHR approximate income 200 JDS
**Refugee 08M**	45	Male	High school	Barber	Married	Almafraq-inside camp	With a wife and nine daughters	Almafraq-inside camp since 2012	Receiving a monthly 50 JDS food coupon and 20JD for each one of a family member by UNCHR<br>Approximate income 320 JDS
**Refugee 09F**	35	Female	High school	Housewife	Married	Almafraq-inside camp	With husband and two sons and two daughters	Almafraq-inside camp since 2013	Are receiving a monthly 50 JDS food coupon and 20JD for each one of a family member by UNCHR<br>Approximate income 220 JDS
**Refugee 09M**	25	Male	Bachelor degree in marketing	Security guard	Married	Almafraq-inside camp	With a wife and one daughter	Almafraq-inside camp since 2013	Receiving a monthly 50 JDS food coupon and 20JD for each one of a family member by UNCHR approximate income 160 JDS
**Refugee 010F**	24	Female	School	Housewife	Married	Almafraq-inside camp	With husband and two sons	Almafraq-inside camp since 2013	Receiving a monthly 50 JDS food coupon and 20JD for each one of a family member by UNCHR<br>Approximate income 180 JDS
**Refugee 010M**	45	Male	High school	Porter	Married	Almafraq-inside camp	With a wife and three sons, and one daughter	Almafraq-inside camp since 2012	Receiving a monthly 50 JDS food coupon and 20JD for each one of a family member by UNCHR approximate income 220 JDS
